# Living Donor Kidney Transplantation in Older Individuals: An Ethical Legal and Psychological Aspects of Transplantation (ELPAT) View

**DOI:** 10.3389/ti.2023.11139

**Published:** 2023-04-21

**Authors:** Aisling E. Courtney, Greg Moorlock, Kristof Van Assche, Lisa Burnapp, Nizam Mamode, Annette Lennerling, Frank J. M. F. Dor

**Affiliations:** ^1^ Regional Nephrology and Transplant Unit, Belfast City Hospital, Belfast, United Kingdom; ^2^ Warwick Medical School, University of Warwick, Coventry, United Kingdom; ^3^ Research Group Personal Rights and Property Rights, University of Antwerp, Antwerp, Belgium; ^4^ NHS Blood and Transplant, Bristol, United Kingdom; ^5^ Department of Surgery, King’s College London, London, United Kingdom; ^6^ The Transplant Centre, Sahlgrenska University Hospital, Gothenburg, Sweden; ^7^ Institute of Health and Care Sciences, The Sahlgrenska Academy, University of Gothenburg, Gothenburg, Sweden; ^8^ Imperial College Renal and Transplant Centre, Hammersmith Hospital, Imperial College Healthcare NHS Trust, London, United Kingdom; ^9^ Department of Surgery and Cancer, Imperial College, London, United Kingdom

**Keywords:** older recipients, access to transplantation, older living donors, age-related bias, inequity in living donor transplantation

## Abstract

Living donor transplantation is the optimal treatment for suitable patients with end-stage kidney disease. There are particular advantages for older individuals in terms of elective surgery, timely transplantation, and early graft function. Yet, despite the superiority of living donor transplantation especially for this cohort, older patients are significantly less likely to access this treatment modality than younger age groups. However, given the changing population demographic in recent decades, there are increasing numbers of older but otherwise healthy individuals with kidney disease who could benefit from living donor transplantation. The complex reasons for this inequity of access are explored, including conscious and unconscious age-related bias by healthcare professionals, concerns relating to older living donors, ethical anxieties related to younger adults donating to aging patients, unwillingness of potential older recipients to consider living donation, and the relevant legislation. There is a legal and moral duty to consider the inequity of access to living donor transplantation, recognising both the potential disparity between chronological and physiological age in older patients, and benefits of this treatment for individuals as well as society.

## Background

Kidney transplantation is the optimal form of renal replacement therapy for suitable patients with end-stage kidney disease (ESKD).

Notably, the demographic profile of the ESKD population is changing, with older patients (≥65 years) representing the fastest growing incident group starting maintenance dialysis therapy in many countries (1–4). Therefore, there is increasing interest in recent years in the outcome of transplantation compared to chronic dialysis treatment in this cohort, as a proportion of older patients will gain significantly in terms of quality and quantity of life with successful kidney transplantation (5–10).

The outcomes of kidney transplantation from living donors (LD) consistently exceed those from deceased donors (DD) in terms of patient and graft survival (11–13). However, the opportunity for kidney transplantation from a living donor is inconsistent across age categories; in the UK, for example, the likelihood of having a LD rather than a DD transplant is almost 90% lower in those aged 65 years or older at time of transplant, compared to young adults (14–17).

The scope of this paper is to explore the inequality of access to living kidney transplantation for the older recipient (defined as >65 years old).

### Advantages of living donor transplantation in older patients

One of the objectives of the current deceased kidney offering scheme in the UK is to maximise the utility of DD organs, in part by preferentially matching kidney life expectancy with recipient life expectancy (18). This mirrors the principles of the Eurotransplant Senior Programme instigated in 1999 (19). Thus if reliant upon deceased donors, older patients are more likely to be offered a kidney from an older donor with associated comorbidity. There is a higher incidence of delayed graft function with such organs (20), requirement for biopsy with attendant hazards, and consequent need for prolonged hospital stay with associated risks of deterioration in functional ability and independence.

There are short-term advantages in receiving a LD organ over a DD organ, particularly in older patients. Transplants (even if coming from older donors) typically work immediately, facilitating early discharge and resumption of normal activities. Additionally, there are particular advantages to elective rather than emergency surgery in older individuals who are more likely to be comorbid than younger patients. Indeed, in some centres there are patients considered suitable *only* for living donor transplantation, where the risk:benefit ratio (considering the combined physiological stress of emergency surgery and a delayed poorly functioning kidney) of a DD transplant is so unfavourable as to be prohibitive. An additional benefit of LD transplantation in the older cohort is the opportunity for minimisation of, or no time on dialysis, i.e., pre-emptive transplantation. Dialysis duration prior to transplantation is arguably the strongest independent modifiable risk factor for kidney transplant outcomes, and this is likely to be of even greater significance in older patients, when decline in functional capacity (including cognitive function) and death on dialysis are accelerated compared to younger age groups (21–27). Thus, older individuals have a more limited window of opportunity for transplantation before the risks are considered excessive.

However as the prevalent age of the ESKD population rises, so does the age of the potential LD pool of siblings, spouses, and friends. There are two areas of potential concern with transplanting from older donors:

• the outcomes for the living donor—is the peri-operative risk unacceptably increased compared to young donors?
• the outcomes for the recipient—is the older transplanted kidney going to provide useful function for an acceptable period of time?

There is widespread acceptance of older DD for older patients with ESKD (19, 28), yet in some centres there is reluctance to consider transplantation from older LD, despite the reassurance of a healthy kidney with no peri-mortal injury.

Given that LD transplantation is the optimal treatment for ESKD in suitable patients, and has particular benefits for older individuals, what are the factors hindering this in practice? Is there inherent age discrimination? Are there ethical and psychosocial barriers within the transplant community that contribute to the discrepancy of access to this healthcare for older individuals?

### Clinical Cases

Table 1 summarises two clinical scenarios with potential LD options for older transplant candidates, and raises challenging questions for healthcare teams.

**TABLE 1:** Potential donors for older patients with end-stage kidney disease.

		Case 1	Case 2
Potential donor	Age	33 years	77 years
	Gender	Male	Male
Potential recipient	Age	71 years	75 years
	Gender	Female	Female
	Cause of ESKD	Obstructive uropathy	Vasculitis
Relationship		Son to mother	Husband to wife
Questions		Is this appropriate?	Is this appropriate?
		How would this offer be considered if he wished to donate to his 5-year old daughter?	What are the alternatives for the potential recipient?

## Systemic Barriers

### Transplant Professionals

#### Younger Donor

The physical risks to a donor are unaltered by the age or health of, or relationship with, the intended recipient. If the potential donor in case 1 was wishing to donate to his 5-year-old daughter, rather than 71-year-old mother, the surgical procedure, recovery, and long-term outcomes from a physical perspective will be identical. Yet, it is likely that few would dispute the appropriateness of proposed donation from the father to the child. The opinion on his donation to his elderly mother however will be considered differently in at least some transplant centres.

This may result from the difference in “value” that society assigns with certain relationships (29) and reflect the influence of the beliefs of the transplant professionals on the perceived “benefit” of his gift. If it is considered of more value to the child than to the parent, the identical physical risks are *relatively* greater when considering donation to the older individual. Is this valid? Is it reasonable that the transplant team makes a judgment call on the value of the transplant outcome for the recipient? (30). And is there account taken of the non-physical benefits to the donor from a successful transplant for the recipient?

Aside from value, there is another potential difference since the obligations that a parent may have to their child are not necessarily replicated in reverse, i.e., a child (even when an adult) does not necessarily have a corresponding duty to their parents. There are arguably certain things that a parent may be morally obliged to do for their children that a child is not obliged to do for their parents. But this cannot provide a compelling difference here, since talk of obligation in LD is itself potentially problematic when it comes to freely given consent. Moreover, given the value of autonomy in LD, it is not obvious that obligation arising from a particular relationship should make a donation more acceptable than an autonomously motivated donation without underlying obligation (30).

Aside from these considerations, it is inevitable that the culture of a transplant centre is influenced by the personal beliefs of its leading professionals (31–33), potentially based on single cases they once experienced (positively and negatively). Additionally, it may reflect a reluctance to change or deal with uncertainty (34). This impacts on the information given to potential donors and recipients, and the enthusiasm with which LD transplantation for older individuals is presented as an option, if at all. Inevitably such differences account, at least in part, for the discrepancy in access to LD transplantation.

#### Older Donor

##### Donor Considerations

Conversely to younger donors, where the concerns relate to long-term rather than short-term risks to health, for people donating at an older age the “long-term” is, by definition, limited but there is a greater potential risk of peri-operative morbidity. It is crucial that transplant teams have confidence in their assessment process in evaluating older volunteers.

An early suboptimal donor outcome, irrespective of age, has a much greater psychological impact on the transplanting team than poor kidney function two or three decades after donation in a younger individual. In the latter scenario, most probably an alternative medical practitioner will then be responsible for care of the donor.

It is likely that such psychological factors, and concern about the possibility of peri-operative events, are contributory to the subconscious assessment of risk. This is reflected in the attitude amongst transplant professionals in Europe towards extended criteria living donors: almost half (43%) reported an upper age limit for LD in one survey, and in another report a third would not consider donation from individuals over 70 years old (31, 33).

Such concerns however are not evidence based. The available literature supports the safety of nephrectomy in older donors assessed according to protocol: 1-year survival in donors aged ≥70 years (in the US from 1990–2010) was 99.5%, comparable to matched controls from the general population (99.1%) (35, 36).

The scenario in case 2 is common: couples that have retirement plans together where the quality of life of the “healthy” partner is substantially negatively impacted by the ESKD of the other. Undoubtedly, giving such individuals the opportunity to donate is transformational for the donor as well as the recipient. The early quality of life reports for the older donor may exceed that of younger contemporaries (37). Imposition of the fears and prejudices of a reluctant, risk-averse transplant team on the decision-making process will impact on achieving the best outcomes for the patient.

##### Recipient Considerations

Another consideration in relation to older potential donors is the likely outcome for the *recipient* in terms of graft function and survival. Younger kidneys are associated with better outcomes. However, there has been a progressive increase in the age of deceased donors over the past four decades. The persistent relative shortage of deceased donor organs, which has driven this, has of course been exacerbated by increasing willingness to consider older healthy patients for transplantation.

It is counter-intuitive therefore not to consider older potential living donors for older recipients, when the alternative is an older DD kidney, or no opportunity of transplantation. The reported outcomes for LD are better than for DD of not only comparable age, but also younger, with the benefits of established good health and function, and avoidance of the physiological catastrophe of death (37–40). The reality for the older recipient is that prolonged survival is not anticipated and therefore a single LD transplant, even from a comparably aged donor, is typically adequate.

### Transplant Patients

Older patients with ESKD have a range of emotional and psychological responses when the possibility of a transplant, particularly from a living donor is discussed (41–43). The seriousness with which this option will be considered will be influenced by the attitude of the transplant team (44)—any reticence will typically translate into a reluctance from the patient to discuss the possibility with potential donors. Common with other specialties, the beliefs of the professional characteristically have a substantial impact on the health choices of the patient.

Even when there is genuine support from the clinicians for LD transplantation, however, the potential recipient often expresses reluctance (45). The feelings of guilt and unworthiness are well described (46), however in relation to the older patient, there are specific issues.

#### Younger Donor

With a younger donor, most commonly a son or daughter, there can be a feeling of “disorder.” In a comparable way as the death of an adult child is felt contrary to the natural cycle of life, so receiving the “gift of life” from a “child” can also feel counterintuitive and inappropriate. Being persuaded that the donor ultimately will also benefit—often this can only be conveyed convincingly by the potential donor—is usually necessary to overcome this barrier. Undoubtedly, considerable advantages to the donor can exist, not just emotionally but often practically in terms of the extended family support. Withholding an opportunity to donate may have a detrimental psychosocial impact on the potential donor (47). When a patient is unwilling to consider younger volunteers, it is important that, rather than simply accepting that there are “no LD options,” the healthcare team enquire about possible volunteers and explore the reasons for decline.

#### Older Donor

When the LD volunteer is older, the reluctance may stem from not wishing to “put the donor at risk.” In this scenario the depth of the emotional relationship may be the most influential factor, along with perhaps the enthusiasm of a partner who has the most to benefit (apart from the recipient themselves), from a successful transplant.

## Ethical Issues

Since LD transplantation is the best treatment option for the patient with ESKD, to state that it is desirable to have more living donation would seem *prima facie* uncontroversial. But there are other perspectives, not just those of the recipient, which must be considered from an ethical perspective and a LD brings additional complexity.

If living donation is considered to bring overall benefit to the potential *donor*, then the argument to provide information about living donation to older patients is stronger. Giving more donors and recipients the opportunity to enjoy the benefits of successful transplantation, with additionally reducing reliance upon the scarce deceased donor pool and the economically draining maintenance dialysis programme, are good things. If living donation is considered not to provide so much benefit to the donor and given that benefits to the donor are possibly lower with older than younger recipients (as inevitably more time-limited), then the argument that living donation should be presented as an option to older recipients is somewhat weaker.

Part of the reason for reluctance to use living donors for older recipients may relate to the principle of utility, with the goal of maximising this for each organ. It could be argued that giving an excellent kidney from a young LD, which may function for at least 20 years in a comparably aged recipient, to an older patient who will only live for another 10 years fails to make full use of that kidney, as 10 years of transplanted kidney function would have been squandered. This argument is flawed because if the living donation never goes ahead in the first place, then all transplanted kidney function is squandered.

Another notable difference between living and deceased donors is that the former can articulate their choice of recipient, which is not possible in deceased donation. The principle of donor autonomy must therefore be in equipoise with utility, in contrast to the situation with deceased donation. Balancing this additionally with healthcare professionals’ paternalistic “protection” can be challenging (48, 49).

## Legal Issues

The right to health, generally defined as “the right to the enjoyment of the highest attainable standard of health,” is enshrined in international human rights law and many national constitutions worldwide (50–52). Arguably, LD transplantation is the best option to achieve this for ESKD patients.

Denying older patients the opportunity to be considered for living kidney transplantation may be a violation of non-discrimination obligations under human rights law. The European Convention on Human Rights stipulates that individuals should not be discriminated against on any ground, including on the basis of age, in the enjoyment of the guaranteed rights such as the right to life and the right to physical integrity. Although health is not explicitly stated in the European Convention, the right to health is expressed in the European Social Charter, which includes a similar anti-discrimination clause.

Importantly, international human rights law has recently emphasised that countries ought to ensure the availability, accessibility, and affordability of healthcare for older persons, and that barriers should be eliminated that deny older persons their rights on an equal basis with other persons (53, 54). More generally, combatting age discrimination in access to healthcare has become a major human rights issue with the adoption in 2015 by the United Nations General Assembly of Sustainable Development Goal 3: Ensure Healthy Lives and Promote Wellbeing for All at All Ages (55).

Although international guidelines on transplantation do not yet explicitly focus on potential discrimination of recipients based on age, they do require provision of equitable access to transplantation services for patients. This means that “all people, whatever their condition or background, must be equally able to be assessed by whatever transplant services are available” (56). Moreover, these guidelines also recommend that organ transplantation services are determined by medical criteria, such as compatibility, medical urgency, and expected outcomes. Age considerations should not in and of themselves therefore be a contraindication to transplantation.

It is also widely accepted in healthcare that for consent to treatment to be valid legally, the patient must be given all relevant information about what the proposed treatment involves, the alternative treatments, and the consequence of not having the treatment. Most countries in Europe have, in their Law on Patient Rights (e.g., Belgium, Netherlands, Sweden—*Patientlag* 2014:821 (57)) or in case law [e.g., for the UK see *Montgomery v. Lanarkshire Health Board* (58)], shifted away from the “reasonable physician standard” towards a “reasonable patient standard” in deciding what counts as relevant information to be disclosed to patients. At least in countries where LD is a well-established treatment option, it can be anticipated that ESKD patients would reasonably expect to be informed of this possibility. Reluctance to present living kidney transplantation as a therapeutic option to this cohort might therefore constitute a breach of legal duty.

It can be argued that LD transplantation cannot be considered an “available” treatment if the patient has a degree of responsibility to “source” a willing donor. However, if donors spontaneously offer to donate they have not necessarily been “sourced” by the recipient. It is of course impossible for anyone to volunteer for something about which they know nothing, so a person has to be made aware by some means, that they can volunteer to be considered as a donor.

In conclusion, older people do not have a legal right to have a living donor transplant, but do have a right to be informed of this possibility where it is an available therapeutic option that would be otherwise be offered to them if they were younger.

## Social Issues

The inequity of access to transplantation and LD transplantation in particular within and between countries, is well recognised, but identifying and then overcoming the barriers is more challenging (59). There are undoubtedly social factors that impact on the ability to access this treatment though published work specifically in relation to older patients is limited; one report suggests there is no association between age and socioeconomic factors (60).

The relatively low LD rate in older age groups, despite obvious advantages suggests that socially this is not an accepted “norm”. Potential older LD and recipients may assume that they are “too old” to be considered and therefore are less likely to volunteer as a donor or be self-active as a potential recipient. Society more broadly has to gain from LD in the older age group with restoration of “normal for age” activities and daily function allowing contribution again to family and societal life. Although the position statement from the European Renal Association-European Dialysis and Transplant Association Descartes Working Group in 2016 stated that elderly patients should be encouraged to consider living donation (61), barriers remain.

## Conclusion

Living donor transplantation offers superior outcomes to both deceased donor transplantation and maintenance dialysis. There are particular advantages for older patients, yet this cohort is significantly less likely to access this treatment option compared to younger age groups. The reasons appear varied and complex. However this inequality cannot always be justified for clinical or ethical reasons, thus there is an age-based inequity of access to transplantation. There is a legal and moral duty to address this with recognition of the potential disparity between chronological and physiological age.

## Data Availability

The original contributions presented in the study are included in the article/supplementary material, further inquiries can be directed to the corresponding author.
